# Characteristics and advances in signaling pathways, cellular communication, cell junctions, and oxidative stress in lymphedema

**DOI:** 10.3389/fcell.2025.1521320

**Published:** 2025-07-22

**Authors:** Qiancheng Zhao, Zhipu Niu, Ying Pan, Yongqi Hao, Yuan Ma, Jiankai Zhao, Jianshi Du, Yiming Yang

**Affiliations:** ^1^ Department of Cell Biology and Medical Genetics, College of Basic Medical Sciences, Jilin University, Changchun, Jilin, China; ^2^ Clinical Medicine, China-Japan Union Hospital of Jilin University, Changchun, Jilin, China; ^3^ Department of Histology and Embryology, College of Basic Medical Sciences, Jilin University, Changchun, Jilin, China; ^4^ College of Basic Medicine, Changchun University of Chinese Medicine, Changchun, Jilin, China; ^5^ Department of Lymphatic and Vascular Surgery, China-Japan Union Hospital of Jilin University, Changchun, Jilin, China; ^6^ Key Laboratory of Lymphatic Surgery Jilin Province, Engineering Laboratory of Lymphatic Surgery, Changchun, Jilin, China

**Keywords:** lymphedema, signaling pathways, inflammation, cell communication, gap junction

## Abstract

Lymphedema is a chronic inflammatory disease without an effective treatment method, and it results in a high disease burden and psychological distress in patients. Although there have been significant advances in targeted therapies, there are still no effective options to treat this refractory disease. In recent years, new advances and breakthroughs have been made in signaling pathways, including RAS/MAPK, PI3K/AKT, VEGF-C/VEGFR-3, HGF/MET, and TGF-β1, which are important for understanding the pathogenesis and disease progression of lymphedema. Mutations in genes encoding cell junctions affect the formation of junctions in lymphatic endothelial cells (LECs), causing abnormal lymphatic valve development and the impairment of lymphatic vessels. A vicious cycle of oxidative stress and chronic inflammation of lymphatic vessels leads to lymphedema. Moreover, the interactions and information communication of T-cell subsets, neutrophils, macrophages, dendritic cells (DCs), and fibroblasts with LECs play equally important roles in the progression of lymphedema. Therefore, this paper summarizes the reported signaling pathways, cell junctions, oxidative stress, and cell communication involved in lymphedema, with the goal of providing ideas and a basis for understanding the pathogenesis, disease progression and targeted therapy of lymphedema. By integrating current findings on signaling dysregulation, cell junctions, and cellular crosstalk, this review provides a conceptual framework for developing multitarget therapeutic strategies to restore lymphatic homeostasis and develop potential therapies for treating lymphedema.

## 1 Background

Lymphedema is a chronic disease of the lymphatic system that is characterized by localized fluid retention, swelling, inflammation, fibrosis, deposition of fatty tissue, and susceptibility to infection (Gousopoulos et al., 2016b; Tashiro et al., 2017; Grada and Phillips, 2017; Bowman and Rockson, 2024). The long-term limitation of limb movement can cause inconveniences and great psychological pressure on patients’ lives, seriously affecting their quality of life (Engel et al., 2003; Khan et al., 2012; Stuiver et al., 2015).

Lymphedema can be classified as either primary lymphedema or secondary lymphedema (Grada and Phillips, 2017; Yamamoto et al., 2015; Pinto et al., 2013) (Table 1). Primary lymphedema results from dysplasia or dysfunction of LECs and their associated vasculature development caused by mutations in genes involved in lymphatic development or function, leading to impaired lymphatic system function (Ferrell, 2002; Karkkainen et al., 2001; Karkkainen et al., 2000). Primary lymphedema is hereditary, as it has been reported that up to 29 genetic abnormalities are related to lymphedema (Michelini et al., 2020). Secondary lymphedema occurs after damage to the lymphatic vessels, such as bacterial or viral infection, cancer treatment, or filarial infection (Cheon et al., 2023; Rockson et al., 2019; Coriddi et al., 2017; Cormier et al., 2010). In secondary lymphedema, filariasis-associated lymphedema is the most common globally, whereas cancer-related lymphedema is more prevalent in Western countries. Global epidemiological patterns reveal significant geographic disparities: filarial lymphedema predominates in certain regions of India and Tanzania, with 250 districts in India alone accounting for more than 40% of the global burden of lymphatic filariasis. Moreover, cancer-related lymphedema affects 21%–28% of breast cancer survivors in Western countries, with the prevalence increasing to 75% among those with gynecological malignancies. (Grada and Phillips, 2017; Walsh et al., 2016; Fimbo et al., 2020; DiSipio et al., 2013; Dayan et al., 2018). Most current lymphedema treatments include massage, manual lymphatic drainage, compression bandages, therapeutic exercise, and dietary intervention (Shimizu et al., 2023). However, there is still a lack of targeted therapies for lymphedema, and there are no treatments based on molecular targets. A few lymphatic venous anastomoses are performed, supplemented by drug therapy, and an effective treatment plan is urgently needed (Lu et al., 2023). Therefore, this review summarizes some of the key signaling pathways involved in lymphedema, the cell junctions between LECs, and cell-to-cell information exchange in lymphedema, with the hope that insights from these studies will ultimately contribute to the development of effective management strategies for lymphedema.

**TABLE 1:** The background information of lymphedema.

Category	Primary lymphedema		Secondary lymphedema		
Pathogenesis	LEC dysplasia/vasculature dysfunction		Injury lymphatic dysfunction		
Key mechanisms	Genic mutation	Heredity	Infection Filariasis (global), virus, bacterial	Cancer treatment: Surgery, radiotherapy (Western prevalence)	Regional hotspots: India (40% global filariasis burden)
Epidemiology	Rare		Filarial: 250 endemic districts in India Cancer-related: 21%–28% breast cancer survivors (West), 75% gynecological malignancies		
Core Therapies	Massage, manual lymphatic drainage, compression bandages, therapeutic exercise, and dietary intervention				

## 2 Physiology of the lymphatic system

The lymphatic system is a tubular network that, together with blood vessels, constitutes the body’s circulatory system and ensures normal physiological activities (Adams and Alitalo, 2007). It is known to be a unidirectional blind-ended system consisting of lymph nodes and lymphatic vessels, including lymphatic capillaries, pre-collecting and collecting vessels (Swartz, 2001; Leak, 1976; Brix et al., 2021). In peripheral tissues, a dense network of nonconventional lymphatic capillaries composed of a single layer of LECs is responsible for pressure-dependent drainage of interstitial fluid, cells, and macromolecules from the interstitial space into the lymphatic system (Bollinger and Partsch, 1985; Castenholz, 1984; Ohtani, 1987; Lynch et al., 2007; Scallan et al., 2022). Special button-like junctions, discontinuous basement membranes, a lack of pericyte coverage, and connections with the surrounding extracellular matrix constitute the high drainage capacity of lymphatic capillaries. Lymph fluid is transported through the anterior collecting ducts containing valves and sporadic smooth muscle cells (SMCs) to the collecting lymphatic vessels (Mandelbrot and Mandelbrot, 1982; Ducoli and Detmar, 2021). The collecting lymphatic vessels have intact basement membranes and “zipper-like” tight junctions and are surrounded by SMCs (Baluk et al., 2007). Both the anterior collecting and collecting lymphatic vessels contain mitral structures or valves that prevent the backflow of lymph nodes and ensure unidirectional lymphatic flow. SMCs, which surround lymphatic vessels, mediate the inward constriction of collecting lymphatic vessels, allowing the upward transport of lymph through multiple lymph nodes. However, there are exceptions (von der Weid, 2013). The contraction of lymphatic vessels lacking SMC coverage, such as pulmonary lymphatic vessels, mainly depends on the force generated by the surrounding tissues (Zawieja, 2009). After ascending through several lymph nodes, the lymph eventually returns to the vasculature through major lymphatic trunks, such as the thoracic duct. Because of this highly structured network, the lymphatic system is well suited to maintain fluid homeostasis. More importantly, the lymphatic system serves as a transport route for antigens and immune cells to initiate immune responses (Liao and von der Weid, 2015). In this context, LECs must follow specific and robust regulatory programs expressing specific lymphatic markers, which ensures not only their correct cell identity and function but also proper maturation and organization of the lymphatic network (Oliver et al., 2005).

## 3 Abnormal signaling pathway of lymphedema

**FIGURE 1:**
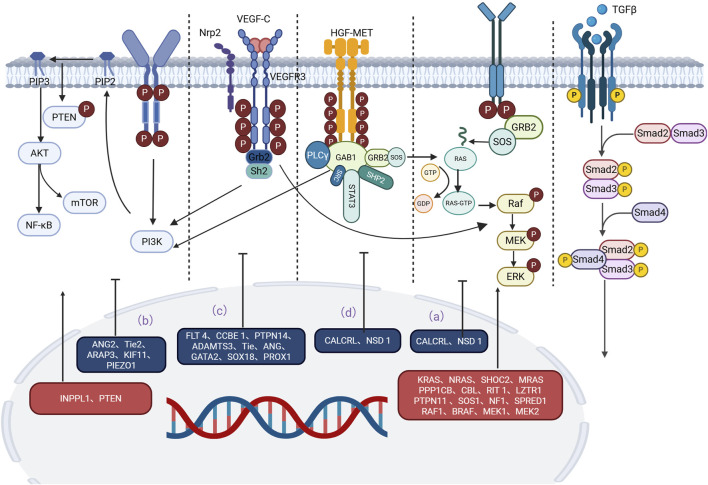
Main signaling pathways involved in the occurrence and development of lymphedema. Mutations in key proteins or regulators in these pathways can lead to overactivation or inhibition of signaling pathways, which can trigger lymphedema. **(a)** RAS‒MAPK pathway: Mutations in CALCRL and NSD1 weaken the RAS‒MAPK signaling pathway. Mutations in KRAS, MEK, SHOC2, MRAS, PP1, SOS1, CBL, RIT1, LZTR1, PTPN11, NF1, SPRED1, RAF1, BRAF, and MEK1/2 often lead to an abnormal MAPK cascade that increases signaling and ultimately leads to lymphedema. **(b)** P13K/AKT pathway: Mutations in INPPL1, ANG2, TIE2, ARAP3, PTEN, KIF11, and PIEZO1 are also strongly associated with lymphedema. **(c)** VEGF-C/VEGFR-3 pathway: Mutations in the LT4, CCBE1, PTPN14, ADAMTS3, TIE, ANG, GATA2, Prox1, Prox1, and SOX18 genes interfere with the normal conduction of the VEGF-C/VEGFR-3 pathway, ultimately leading to lymphedema. **(d)** HGF/MET pathway: Mutations in HGF and MET disrupt signal transduction in the HGF/MET system and lead to lymphedema. An in-depth study of these signaling pathways is expected to provide new targets for the prevention and treatment of lymphedema.

A growing body of evidence has revealed important signaling pathways involved in lymphedema-related mechanisms. Signaling pathways such as the RAS/MAPK pathway, the PI3K/AKT pathway, the TGF-β1 pathway, and the HGF/MET signaling pathway have been confirmed to play important roles in the development and progression of lymphedema (Visser et al., 2012; Tidyman and Rauen, 2008; Fritz-Six et al., 2008; Agollah et al., 2014; Korhonen et al., 2022; Suzuki et al., 2022; Finegold et al., 2008; Koksharova et al., 2024; Alpaslan et al., 2024) (Table 2). The effects of these pathways are closely correlated with the upstream VEGF-C/VEGFR-3 signaling axis. Moreover, some of the less reported signaling pathways, such as the S1P, Notch1, NF-κB, EphrinB2/EphB4, Dchs1 and Fat4 signaling pathways, are similarly discussed below. As information on these pathways in relation to lymphedema remains limited, they provide new perspectives for future research and potential new targets for lymphedema treatment. The exchange of cell-to-cell information between different gene mutations, regulatory molecules, and key components of pathways is involved in the progression of lymphedema. According to the classification of signaling pathways, while introducing regulatory molecules and gene mutations related to lymphedema, we generated tables and pictures for reference (Figure 1).

**TABLE 2:** Genes involved in the lymphedema signaling pathway.

Signaling pathway	Genes	Experimental model	Mechanisms of action	Types of lymphedema	Data sources
RAS/MAPK	AM (Fritz-Six et al., 2008) CALCRL (Fritz-Six et al., 2008) RAMP2 (Fritz-Six et al., 2008)	Knockout mice LECs HUVECs	Necessary for murine lymphatic vascular development Enhanced activation of ERK signal transduction	Primary	Human Mice
	KRAS (Sheppard et al., 2023)	HDLECs Zebrafish	variants activate the RAS/MAPK pathway leading to lymphatic dysplasia and edema	Primary	Human Zebrafish
	NRAS (Carta et al., 2006; Ekvall et al., 2015) CBL (Hanson et al., 2014; Rodriguez-Viciana et al., 2006; Young et al., 2018; Brand et al., 2014; Castel et al., 2019) LZTR1 (Castel et al., 2019; Motta et al., 2019) PTPN11 (Bonetti et al., 2022; Martinelli et al., 2010; Tartaglia et al., 2002) SOS1 (Tidyman and Rauen, 2009; Roberts et al., 2007) SPRED1 (Cirstea et al., 2010) NF1 (Cirstea et al., 2010) RAF1 (Cirstea et al., 2010) BRAF (Cirstea et al., 2010) MEK1 (Cirstea et al., 2010) MEK2 (Cirstea et al., 2010)	NS patients	Mutations cause disruption of the Ras/MAPK signaling pathway	Primary	Human
	SHOC2 (Hanson et al., 2014; Rodriguez-Viciana et al., 2006; Young et al., 2018; Brand et al., 2014; Castel et al., 2019) MRAS (Hanson et al., 2014; Rodriguez-Viciana et al., 2006; Young et al., 2018; Brand et al., 2014; Castel et al., 2019) PP1 (Hanson et al., 2014; Rodriguez-Viciana et al., 2006; Young et al., 2018; Brand et al., 2014; Castel et al., 2019)	HEK293 DLD-1	Mutations in MRAS, SHOC2, and PP1 contribute directly to NS by enhancing formation of a ternary complex	Primary	Human
	RIT1 (Castel et al., 2019; Motta et al., 2019)	Mutant mouse model HEK293	Mutations cause disruption of the Ras/MAPK signaling pathway	Primary	Human Mice
	NSD1 (Visser et al., 2012)	SoS patients	Mutations cause disruption of the Ras/MAPK signaling pathway	Primary	Human
PI3K/AKT	INPPL1 (Agollah et al., 2014)	Lymphatic disease family HDLECs	Negative regulation of the PI3K signaling pathway, whose mutation is associated with lymphatic dysfunction	Primary	Human
	ANG2 (Korhonen et al., 2022) Tie2 (Korhonen et al., 2022)	Knockout mice	Gene deletion leads to abnormal development and function of embryonic lymphatic vessels, resulting in lymphatic dysfunction	Primary	Human Mice
	ARAP3 (Kartopawiro et al., 2014)	Mouse model Zebrafish	Lymphatic vessel development is essential	Primary	Mice Zebrafish
	PTEN (Goberdhan and Wilson, 2003; Kataru et al., 2021)	Knockout mice	The loss of PTEN leads to increased phosphorylation of AKT, thereby increasing LEC proliferation and differentiation	Primary	Mice
	KIF11 (Ostergaard et al., 2012) PIEZO1 (Fotiou et al., 2015)	MLCRD Patients Genetic and data analysis	Both KIF11 and PIEZO1 can activate the PI3K/AKT signaling pathway and increase cytoplasmic Ca2+ concentration Mutations affect the development of lymphatic structures	Primary	Human
VEGF-C/VEGFR-3	FLT 4 (Gordon et al., 2013; Irrthum et al., 2000)	Several animal models	Plays an important role in lymphatic vessel formation; mutations prevent phosphorylation	Primary	Several animal models
	CCBE 1 (Brouillard et al., 2017; Bos et al., 2011; Au et al., 2010) ADAMTS3 (Brouillard et al., 2017; Bos et al., 2011; Au et al., 2010)	Hennekam syndrome patients Mouse model	Mutations can cause lymphatic abnormalities and even death	Primary	Human Mice
	PTPN14 (Brouillard et al., 2017; Bos et al., 2011; Au et al., 2010)	Mouse model	Lymphatic hyperplasia with lymphoedema following genetic defect	Primary	Human Mice
	ANG2 (Korhonen et al., 2022) Tie2 (Korhonen et al., 2022)	Knockout mice	Gene deletion leads to abnormal development and function of embryonic lymphatic vessels, resulting in lymphatic dysfunction	Primary	Human Mice
	GATA2 (Kazenwadel et al., 2015; Mansour et al., 2010)	Emberger syndrome patients Mouse model	It is required for lymphatic vessel valve development and maintenance	Primary	Huamn Mice
	SOX18 (Irrthum et al., 2003; Brouillard et al., 2014)	Clinical patients	Mutations can cause lymphatic dysfunction and lymphoedema	Primary	Human
	Prox1 (Kazenwadel et al., 2015; Mansour et al., 2010; Wigle and Oliver, 1999)	Knockout mice LECs	Essential for lymphatic system development Critical for the differentiation of LECs	Primary	Human Mice
HGF/MET	HGF (Finegold et al., 2008) MET (Finegold et al., 2008)	Lymphedema patient Mouse model LECs	Promotes LEC proliferation, migration and tube formation Promotes lymphatic vessel formation in mice	Primary Secondary	Human Mice
TGF-β1	TGF-β1 (Sano et al., 2020) Smad (Sano et al., 2020)	Mouse model Lymphedemapatients	TGFB1 is a key regulator of accelerated fibrosis while inhibiting lymphangiogenesis	Secondary	Human Mice
NF-κB	IKBKG (Roberts et al., 2010)	Lymphedema patient	Affects activation of the NF-κB signaling pathway, and defects in this pathway may lead to lymphatic vessel dysfunction	Primary	Human
	STAT3 (Bowman and Rockson, 2024; Satapathy et al., 2006; Levy and Lee, 2002)	Lymphocyte Mouse model	Role in the inflammatory and fibrotic processes of lymphedema	Secondary	Human Mice
	NLRP3 (Zhou et al., 2023)	Mouse model	Promotes macrophage M2 polarization	Secondary	Mice
	TLR4 (Li et al., 2024) VEGF-C (Li et al., 2024)	Mouse model	Promote lymphatic angiogenesis to reduce lymphedema	Secondary	Mice
EphrinB2/EphB4	EphrinB2 (Frye et al., 2020) EphB4 (Frye et al., 2020) CLDN5 (Frye et al., 2020)	Mouse model HDLECSs	LECs connections are required and selectively regulate lymphatic vessel permeability and function	Primary Secondary	Human Mice
Dchs1 and Fat4	Dchs1 (Pujol et al., 2017) Fat4 (Pujol et al., 2017)	Knockout mice Hennekam patients	Controls endothelial cell polarization during lymphatic valve morphogenesis	Primary	Human Mice
Notch1	Notch1 (Jannaway et al., 2023) DLL4 (Jannaway et al., 2023)	Lymphedema patients Mouse model	Functional blockade can lead to lymphedema disorders	Primary	Human Mice
S1P	S1P (Kim et al., 2023)	Mouse model LECs	Signaling abnormalities exacerbate lymphatic dysfunction and tissue inflammation	Secondary	Human Mice
TRML1	TRPML1 (Yang et al., 2024) AQPs (Yang et al., 2024)	Mouse model HUVECs LECs	Alteration of cell permeability through regulation of AQPs localization triggers lymphedema with chronic inflammation	Secondary	Human Mice
Wnt/β-catenin	Wnt (Cha et al., 2018) β-catenin (Cha et al., 2018)	Knockout mice HLECs LECs	Plays a key role in the development of both lymphatic vessels and valves	Primary	Human Mice

### 3.1 RAS/MAPK signaling pathways

The RAS/MAPK pathway is closely associated with lymphedema development. For example, Noonan syndrome (NS), cardio-facio-cutaneous (CFC) syndrome, and Sotos syndrome are all lymphedema syndromes associated with RAS/MAPK pathway abnormalities (Visser et al., 2012; Tidyman and Rauen, 2008). Many regulatory molecules and key components play important roles in the progression of lymphedema. For example, mutation or deletion of AM, CALCRL and RAMP2 leads to a lack of normal activation of the MAPK/ERK signaling pathway, resulting in reduced LEC proliferation, hypoplastic lymphatic vessels, and ultimately interstitial lymphedema (Fritz-Six et al., 2008). Sheppard SE et al. reported that the expression of activated KRAS variants increased the phosphorylation of ERK and increased MAPK signaling in human dermal lymphatic endothelial cells (HDLECs), leading to lymphatic dysplasia and edema (Sheppard et al., 2023). In the following text, we address the existing reports of NS, CFC, and Sotos syndrome with primary lymphedema separately.

NS, caused by germline pathogenic variants of genes within the RAS/MAPK signaling pathway, is characterized mainly by symptoms of lymphatic dysplasia and abnormalities of the lymphatic system (Sleutjes et al., 2022). NS patient species were also detected for germline mutations in both KRAS and NRAS, although in a small proportion (Carta et al., 2006; Ekvall et al., 2015). Most genes involved in NS encode proteins indispensable to the RAS/MAPK pathway, and pathogenic mutations often increase signaling through this pathway (Schubbert et al., 2007). For example, the gene encoding the SHOC2–MRAS–PP1 complex is a positive regulator of the RAS-ERK signaling cascade, and the gene encoding CBL can negatively regulate intracellular signaling downstream of the receptor tyrosine kinase. Mutations in the genes encoding SHOC2, MRAS, PP1 and CBL can cause symptoms similar to those of NS (Hanson et al., 2014; Rodriguez-Viciana et al., 2006; Young et al., 2018; Brand et al., 2014; Castel et al., 2019). Moreover, NS is closely associated with aberrant mutations in RIT1, LZTR1, PTPN11 and SOS1, which regulate the RAS/MAPK pathway. Castel P and Motta M et al. reported that the RIT1 and LZTR1 genes regulate the progression of severe bilateral lower limb lymphedema in adolescent patients through abnormal activation of the RAS/MAPK pathway (Castel et al., 2019; Motta et al., 2019). Tartaglia M et al. reported that 50% of NS cases are caused by missense gain-of-function mutations in PTPN11, which affect the domain of SHP2 and thus the signaling in the RAS/MAPK pathway (Maheshwari et al., 2002; Tartaglia et al., 2001). Missense mutations in SOS1, which occur in approximately 13% of cases of NS, increase RAS activity and subsequently enhance RAS/MAPK signaling pathway transduction by disrupting the autoinhibition of RAS-GEF activity (Tidyman and Rauen, 2009; Roberts et al., 2007). In addition, a report by Cirstea IC reported that germline mutations in the RAS regulatory factors NF1 and SPRED1, as well as downstream signaling factors such as RAF1, BRAF, MEK1, and MEK2, are involved. These mutations typically lead to abnormalities in the MAPK cascade, resulting in increased signal flow (Cirstea et al., 2010). Studies and case reports of lymphatic abnormalities in NS patients caused by RRAS2 gene mutations are still rare and are valuable for further investigations. In conclusion, NS mutations in patients, such as those in KRAS, NRAS, SHOC2, MRAS, PP1, CBL, RIT1, LZTR1, PTPN11, SOS1, NF1, SPRED1, RAF1, BRAF, MEK1, and MEK2, deserve further investigation of the specific mechanism of action in primary lymphedema.

Studies of the RAS/MAPK pathway in CFC and Sotos syndrome remain scarce. The NSD1 gene product is postulated to be a corepressor protein of growth-promoting genes. Sotos syndrome is a childhood overgrowth syndrome caused by haploinsufficiency of the NSD1 gene (Kurotaki et al., 2002). McClelland J reported the presence of primary lymphedema in female patients with Sotos syndrome caused by NSD1 internal mutations (McClelland et al., 2016). Genetic mutations in NSD1 are accompanied by lower phosphorylation levels of the MAPK kinase (Visser et al., 2012). Similarly, CFC, caused by genetic mutations in BRAF, MEK1/2, HRAS, and KRAS that lead to altered signaling by the RAS/MAPK pathway, is a very rare sporadic disease accompanied by symptoms of primary lymphedema (Morcaldi et al., 2015).

Currently, abnormalities in the RAS/MAPK pathway are reflected in the different syndromes associated with primary lymphedema, and more genes remain to be discovered. Moreover, studying the abnormal manifestations of the RAS/MAPK signaling pathway in primary lymphedema is highly valuable for the study of pathogenesis and for the further development of therapeutic methods.

### 3.2 PI3K/AKT signaling pathway

The PI3K/AKT signaling pathway plays a critical role in many aspects of cell growth, proliferation, survival, metabolism, and migration and is one of the core pathways regulating cell function (Sheng et al., 2003; Zhao et al., 2004). Both VEGF-C/VEGFR-3 signaling and HGF/MET signaling can activate the PI3K/AKT pathway. Similar to the MAPK/ERK signaling pathway, the activation of the PI3K/AKT signaling pathway plays a crucial role in the development of lymphedema. For example, Coso S et al. reported that the activation of PI3K/AKT by VEGF-C/VEGFR-3 leads to the phosphorylation of P70S6K, eNOS, PLCγ1, and ERK1/2, thus participating in the formation of lymphatic vessels (Coso et al., 2012). Yang Y et al. reported that VE-cadherin regulates AKT signaling, which is involved in lymphatic valve formation and maintenance (Yang et al., 2019). The role of VEGF-C/VEGFR-3 signaling and HGF/MET signaling in the activation of components of the PI3K/AKT signaling pathway in the pathogenesis of lymphedema should be further explored.

Many regulatory molecules, such as SHIP-2, VEGF-C, and H2S, are able to modulate the PI3K/AKT signaling pathway to play important roles in lymphedema. Agollah GD et al. reported that mutations in the INPPL1 gene encoding SHIP-2 lead to hyperactivation of PI3K/AKT signaling, which is negatively regulated by SHIP-2 to mediate various forms of lymphedema, due to reduced LEC proliferation, adhesion, migration, and lumen formation (Agollah et al., 2014). Korhonen EA et al. reported that the activation of Ang2/Tie/PI3K signaling in LECs induced by VEGF-C promotes lymphatic vessel formation, which provides further investigation and potential therapeutic means for the targeted treatment of secondary lymphedema (Korhonen et al., 2022). Suzuki J et al. reported that H2S activates the PI3K/AKT signaling pathway in LECs, thus improving secondary lymphedema (Suzuki et al., 2022). Some of these regulatory molecules are gene expression products of LECs, and some are endogenous molecules that regulate PI3K/AKT signaling and are involved in the progression of lymphedema.

Genetic mutations in PIK3CA, ARAP3, PIEZO1, and PTEN also play important roles in the PI3K/AKT signaling pathway in the development of lymphedema. Mutations in genes encoding proteins that regulate the PI3K/AKT signaling pathway can also contribute to the progression of lymphedema. The PI3K/AKT pathway can be activated by two auxiliary proteins: KIF11 and PIEZO1. KIF11 activates PI3KA and increases the cytoplasmic Ca^2+^ concentration (Bonetti et al., 2022). Ostergaard P noted that KIF11 mutations are associated with congenitallymphedema (Ostergaard et al., 2012). Fotiou E noted that novel mutations in PIEZO1 can also lead to primary lymphedema (Fotiou et al., 2015). Furthermore, Krugmann S et al. reported that ARAP3 is also a genuine effector of the PI3K signaling system (Krugmann et al., 2002). Moreover, Kartopawiro J et al. reported that the deletion of ARAP3 caused subcutaneous lymphatic vascular dysplasia and hereditary lymphedema (Kartopawiro et al., 2014). In comparison, Kataru RP and colleagues reported that in a PTEN knockout mouse model, the inhibition of PTEN promoted PI3K/AKT signaling in LECs, thereby increasing LECs proliferation. This provides a potential target for improving lymphatic growth and function (Goberdhan and Wilson, 2003; Kataru et al., 2021). Gene mutations affecting the PI3K/AKT signaling pathway still deserve further investigation and discussion.

Interestingly, there is crosstalk between the PI3K/AKT pathway and the MAPK/ERK pathway in RAS and PI3KA. PI3KA can also be activated by RAS, connecting the RAS/MAPK and PI3K/AKT pathways (Bui and Hong, 2020). Rodriguez-Viciana P et al. reported that PI3K activation by RAS is correlated with direct binding between RAS and p110 (Rodriguez-Viciana et al., 1994; Chan et al., 2002). Zimmermann S et al. reported that the phosphorylation of RAF by AKT inhibited the RAS–RAF–MEK–ERK cascade (Zimmermann et al., 1999). The interaction of the PI3K/AKT pathway and the MAPK/ERK pathway in lymphedema is important for mechanistic studies. The links between these signaling pathways should be further discussed.

### 3.3 VEGF-C/VEGFR-3 signaling pathway

VEGF-C and VEGF-D bind VEGFR3 on the surface of LECs and induce lymphatic proliferation, migration, and survival by activating the intracellular PI3K-AKT and MAPK-ERK signaling pathways (Kuonqui et al., 2023). Many studies have confirmed that mutations in the FLT4 gene encoding VEGFR3 cause Milroy’s disease, an inherited primary lymphedema (Gordon et al., 2013; Irrthum et al., 2000). Many regulatory proteins are involved in lymphedema and are regulated by the VEGF-C/VEGFR-3 axis. Mutations in CCBE1, PTPN14, and ADAMTS3 can lead to abnormal VEGF-C/VEGFR-3 signaling, causing lymphedema (Brouillard et al., 2017; Bos et al., 2011; Au et al., 2010). Korhonen et al. reported that deletion of the Tie receptor or blockade of Ang2 also reduced lymphangiogenesis induced by VEGF-C (Korhonen et al., 2022). Several transcription factors and gene mutations are similarly involved in regulating the VEGF-C/VEGFR-3 axis to influence the development of lymphedema. Mutations in the GATA2 gene, the transcription factor controlling Prox1 and FOXC2 expression, cause primary lymphedema with myelodysplasia (Kazenwadel et al., 2015; Mansour et al., 2010). Prox1 is a key transcription factor in the differentiation of LECs. Prox1 can repress BEC-specific markers and upregulate LEC-specific genes. Sunju Lee et al. reported that blood vascular endothelial cells (BECs) in the main vein are able to migrate and form primitive lymphatic vessels and that both VEGF-C and Prox1 are indispensable regulators of this process, accompanied by the suppression of BEC-specific markers and an increase in the levels of LEC-specific genes, such as VEGFR-3 and FGFR-3 (Lee et al., 2009). In mice, Prox1-deficient mice are unable to form a lymphatic system resulting in severe edema and death (Wigle and Oliver, 1999). In addition, abnormal lymphatic leakage from lymphatic vessels promoted by Prox1 haploinsufficiency contributes to adult-onset obesity (Harvey et al., 2005). Lymphatic leakage also creates an immunosuppressive environment in Prox1^+/−^ mice defective in the Prox1 gene (Herrada et al., 2022). Mutations in SOX18 may indirectly regulate VEGFR3 expression by affecting Prox1, resulting in lymphedema (Irrthum et al., 2003; Brouillard et al., 2014). Moreover, the transcription factors FOXC2 and NFATc1 are both involved in lymphatic valve development and the maturation of the lymphatic system, which may provide potential therapeutic means for the treatment of lymphedema (Norrmén et al., 2009). VEGFR3 was once thought to maintain button junctions, but recent studies have shown that it is not required throughout the life cycle after the formation of button junctions (Jannaway et al., 2023). The VEGF-C/VEGFR-3 signaling pathway is important in the mechanism of lymphedema and in the formation of normal lymphatic vessels.

### 3.4 HGF/MET signaling pathway

Currently, an increasing number of case studies support the potential role of HGF mutations in primary and secondary lymphedema. By sequencing the HGF and MET genes in patients with lymphedema and patients with secondary lymphedema after cancer treatment, Finegold DN et al. reported that four HGF mutations and two MET gene mutations were closely associated with multiple types of lymphedema, which may provide therapeutic targets for lymphedema (Finegold et al., 2008). Koksharova G et al. reported a close association between HGF gene mutation and clinical symptoms of lymphedema. Genetic mutations in HGF are also involved in the pathogenesis of lymphedema (Koksharova et al., 2024). In a study of 770 patients, Alpaslan M et al. reported that mutations in the HGF gene result in mRNA degradation or impaired protein function, which impairs the activation of the AKT and ERK1/2 pathways, leading to primary lymphedema, mainly in the lower limbs, with the onset of the disease extending from childhood to adulthood (Alpaslan et al., 2024). Regulatory molecules of the HGF/MET signaling pathway are equally able to participate in the progression of lymphedema. Bonetti G reported that CBL and PTPN11 are also involved in MET signal transduction. Among them, PTPN11 can activate RAS and AKT, thus acting on both the AKT/PI3K pathway and the RAS/MAPK pathway. Functional mutations in CBL and PTPN11 can cause NS with lymphedema (Bonetti et al., 2022; Martinelli et al., 2010; Tartaglia et al., 2002). The HGF/MET signaling pathway plays an important role in the pathogenesis of lymphedema, which is closely related to the interaction of RAS/M APK signaling and PI3K/AKT signaling.

### 3.5 TGF-β1 signaling pathway

The activation of the TGF-β1 signaling pathway has been identified as a pivotal factor in the progression of fibrosis in lymphedema. Sano M et al. utilized a rat model to elucidate the onset of dermal fibrosis with activation of the TGF-β1/Smad signaling pathway, thereby providing the first evidence of the role of this pathway in the development of secondary lymphedema in humans (Sano et al., 2020). Increased expression and abnormal activation of TGF-β1 promote skin fibrosis in patients with secondary lower extremity lymphedema (Di et al., 2016). Recent studies have shown that TGF-β1-activated fibroblast subpopulations are closely associated with the disease stage and fibrosis progression of lymphedema in human and mouse lymphedema tissues (Will et al., 2024a). As some research findings suggest, dysregulated TGF-β1 promotes extracellular matrix deposition in patients with breast cancer-related lymphedema (BCRL) and in mouse models (Baik et al., 2022). The inhibition of TGF-β1, a multifaceted mediator of fibrosis and extracellular matrix deposition, has been shown to reduce T-cell inflammatory infiltration (Avraham et al., 2010). This finding is further supported by the observations of Sun D et al., who reported that TGF-β1 is overexpressed in the skin of patients with symptomatic secondary lymphedema. In addition, studies have demonstrated that its inhibition *in vivo* can increase neolymphangiogenesis and alleviate lymphedema in a rodent tail model (Di et al., 2016; Avraham et al., 2010; Yan et al., 2011). Furthermore, Baik JE et al. reported that TGF-β1 is an essential regulator of ECM deposition in secondary lymphedema and that inhibition of this response is a promising means of treating lymphedema (Baik et al., 2022). Notably, TGF-β1 also possesses immunosuppressive properties, which could contribute to chronic inflammation secondary to lymphedema. TGF-β1 influences immunoinflammation by modulating the Th17-Treg balance (Yoshimura and Muto, 2011). It has also been shown to inhibit inflammatory signaling pathways such as the NF-κB pathway and reduce the expression of inflammatory factors and chemokines, including IL-6, IFN-γ, and RANTES (Shiou et al., 2013; Dai et al., 2011). LECs has been observed to secrete factors such as TGF-β1, which functions to inhibit DC maturation, thereby reducing inflammation in lymphoid tissues (Christiansen et al., 2016). However, Avraham T’s study revealed that the inhibition of TGF-β function reduces chronic inflammation, thereby rendering the role of TGF-β in secondary lymphedema more complex and elusive. Consequently, the inhibition of TGF-β1 has emerged as a promising treatment for secondary lymphedema. The role of TGF-β1 in the development and progression of lymphedema warrants further exploration, and we anticipate providing novel concepts and methodologies for targeting TGF-β1 in the treatment of lymphedema.

### 3.6 Other signaling pathways

#### 3.6.1 NF-κB signaling pathway

Roberts CML et al. reported that the occurrence of lymphedema in a 6-year-old boy was closely related to the mutation of the IKBKG gene, which encodes the NEMO protein (IKK γ) to regulate the activation of the NF-κB signaling pathway (Roberts et al., 2010). The chronic inflammatory state and fibrosis in lymphedema are strongly associated with STAT3 and NF-κB (Bowman and Rockson, 2024). A study revealed elevated IL-6 levels in secondary filarial lymphedema (Satapathy et al., 2006). IL-6 is able to hyperactivate downstream STAT3 (Levy and Lee, 2002). In addition, there is a synergistic interaction between STAT3 and NF-κB to produce various inflammatory cytokines. STAT3 and NF-κB may undergo positive feedback amplification to maintain the chronic inflammatory state of lymphedema (Hirano, 2021). NF-κB plays an important role in different types of secondary lymphedema. Zhou Z et al. reported that Substance P promoted macrophage M2 macrophage polarization by regulating the NF-κB/NLRP3 signaling pathway, thus alleviating secondary lymphedema (Zhou et al., 2023). In addition, Yang C reported for the first time that the NF-κB inhibitor bortezomib can relieve limb lymphedema in patients with multiple myeloma (Yang et al., 2021a). In secondary lymphedema associated with lymphatic filariasis infection, Babu S et al. reported that TLR2 and TLR9 are involved in inducing the activation of the MAPK and NF-κB pathways to promote the pathological process of lymphedema (Babu et al., 2011). Interestingly, NF-κB plays an important role in lymphatic vessel formation, which may provide new ideas and directions for the subsequent treatment of lymphedema. Li et al. reported that type II arabinogalactan (CAPW-1) could activate the TLR4/NF-κB/VEGF-C signaling pathway in LECs to promote lymphatic angiogenesis and reduce edema in mice with secondary lymphedema (Li et al., 2024). In summary, inhibition of the inflammatory cell NF-κB pathway is important in the treatment of secondary lymphedema. In addition, the activation of NF-κB in LECs is also important in the development and progression of lymphedema. This seems to be a bidirectional issue that requires further investigation in the future.

#### 3.6.2 EphrinB2/EphB4 signaling pathway

The diminished signaling between EphrinB2 and EphB4 in lymph vessels mirrors the heightened vessel leakage evident in response to bacterial infections and primary lymphedema. This phenomenon is associated with a decrease in CLDN5 levels at LEC junctions, a consequence of the loss of EphrinB2-EphB4 signaling (Frye et al., 2020). The EphrinB2/EphB4 signaling pathway has been identified as a promising target for the treatment of lymphedema and related diseases. The specific signaling pathways of EphrinB2-EphB4 in LECs are not yet fully understood. However, the activation of EphB4 receptors or their downstream signaling pathways, along with the regulation of signaling between these two proteins, may provide new therapeutic approaches through the development of drugs that increase the integrity of LECs.

#### 3.6.3 Dchs1 and Fat4 signaling pathways

Abnormal valve development in mice with defective Dchs1 and Fat4 genes causes blocked lymphatic fluid flow and triggers primary lymphedema (Pujol et al., 2017). This study highlights the possibility that abnormal signaling pathways in related genetic disorders may lead to lymphatic system disorders.

#### 3.6.4 Notch1 signaling pathway

Notch1 has been shown to engage in crosstalk with the VEGF signaling pathway, thereby influencing lymphatic vessel development and function. In their seminal study, Michelini S et al. advanced a compelling proposition regarding the most common genetic variant associated with genetic susceptibility to Notch1 lymphedema (Michelini et al., 2021). LECs lacking Notch1 signaling result in abnormal enlargement and formation of lymphatic vessels. Jannaway M et al. reported that forced activation of Notch1 signaling in the VEGFR3/Notch1 signaling axis in the absence of VEGFR3 restored lymphatic capillary button junction formation and interstitial fluid uptake (Jannaway et al., 2023). Upregulation of Notch1 signaling ameliorates VEGFR3 deficiency and can be used as a potential therapeutic approach for treating lymphedema (Jannaway et al., 2023). Notch1 signaling may play an important role in the prevention or treatment of acquired lymphedema. Tian W et al. reported that patients with acquired lymphedema have elevated levels of the proinflammatory mediator leukotriene B4 (LTB4), which inhibits VEGFR3 and Notch signaling involved in lymphedema. In addition, Notch1 knockout mice are insensitive to the therapeutic effects of LTB4 antagonists, which further supports the important role of Notch1 signaling in LTB4 regulation of lymphedema (Tian et al., 2017). Piezo1 is able to downregulate downstream Notch1. Choi D et al. reported that activation of Piezo1 or the use of the Piezo1 agonist Yoda1 effectively inhibited the development of postoperative lymphedema and enhanced lymphoid sprout growth (Choi et al., 2022). Interestingly, whether Notch1 signaling is downregulated or upregulated in the treatment of lymphedema, which may result from differences in Notch1 upstream signaling, remains a question worth exploring. In conclusion, the Notch1 signaling pathway can be targeted for the treatment of lymphedema. The development of drugs related to the Notch1 signaling pathway may be beneficial for the treatment of lymphedema.

#### 3.6.5 S1P signaling pathway

In a study by Kim D et al., the inhibition of the S1P signaling pathway was confirmed to increase the expression of P-selectin in LECs, which relieved the symptoms of secondary lymphedema (Kim et al., 2023). Currently, research on the pathogenesis of the S1P signaling pathway in lymphedema is still scarce. Focusing on the link between S1P signaling and other pathways and its role in disease progression provides new insights and treatments for lymphedema.

#### 3.6.6 Wnt/β-catenin signaling pathway

FOXC2 and GATA2 are also crucial for the differentiation of lymphatic valve endothelial cells. Oscillatory shear stress (OSS) enhances Wnt/β-catenin signaling in cultured LECs, inducing the expression of lymphedema-associated transcription factors GATA2 and FOXC2 (Choi et al., 2016; Cha et al., 2016).PROX1 is a key transcription factor in LECs differentiation.Further studies by Cha et al. revealed that PROX1 interacts with β-catenin and TCF7L1 to promote the expression of FOXC2 and GATA2 (Cha et al., 2018).Research on the Wnt/β-catenin signaling pathway not only reveals the molecular mechanisms involved in LEC differentiation but also provides new ideas and directions for the molecular-level treatment of lymphedema.

#### 3.6.7 TRPML1/AQPs signaling pathway

Cell permeability is considered one of the potential pathophysiological mechanisms of lymphedema. In particular, the dynamic relocalization of aquaporins (AQPs) within cells significantly affects cell permeability (Conner et al., 2013). A recent study demonstrated that TRPML1 induces the accumulation of AQP2 at the apical surface of cells while disassembling the actin cytoskeleton, significantly increasing cell permeability and enhancing the response of cells to hypoosmotic stimulation in terms of water flux (Scorza et al., 2023). Our lab’s latest research revealed that TRPML1 regulates the localization of AQP3 and AQP5 on the cell membrane in human lymphatic endothelial cells (HLECs), leading to increased cell permeability, promoting the development of lymphedema, and accompanying chronic inflammatory responses (Yang et al., 2024). These findings suggest that TRPML1 may be an important trigger for lymphedema. With further research into the functions of TRPML1 and aquaporins, new molecular therapeutic targets for secondary lymphedema are expected to emerge in the future.

## 4 Cellular communication in lymphedema

**FIGURE 2:**
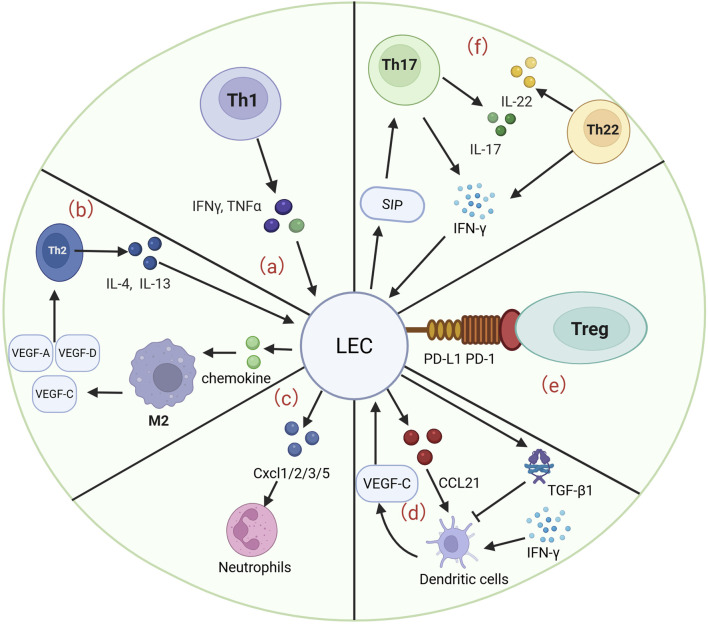
LECs interact with many cells to participate in the development and progression of lymphedema. **(a)** Th1 cells are able to release IFN-γ and TNF-α to act on LECs, which are involved in the pathological process of lymphedema and the progression of fibrosis. **(b)** Th2 cells are able to release IL-4 and IL-13 to promote the progression of LEC apoptosis. LECs are able to act on Th2 cells by releasing chemokines that act on M2 macrophages to induce the production of VEGF-A/C/D, enhancing lymphangiogenesis and fibrosis. **(c)** LECs were able to release CXCL 1/2/3/5 to regulate neutrophil activity and enhance the infiltration of inflammatory cells in a murine lymphedema model. **(d)** VEGF-C secreted by DCs is able to drive CCL21 secretion by LECs, which guides DCs into lymphatic vessels. LECs are able to release TGF-β1 to inhibit DC maturation and subsequently reduce the inflammatory response. **(e)** LECs are able to inhibit the inflammatory progression of lymphedema by binding PD-L1 to PD-1 on Tregs. **(f)** S1P gradient in which LECs are able to recruit Th17 cells. Th17 cells in lymphedema tissue secrete IL-17 and IFN-γ. Similarly, Th22 cells are able to release high levels of IL-22 and IFN-γ, both of which are involved in the progression of lymphedema.

Different subsets of T cells, including Th 1, Th 2, Th 17, Th 22 and Treg cells, play important roles in the pathological process and pathogenesis of lymphedema. The interaction of different subpopulations with LECs is important for a deeper understanding of lymphedema. Moreover, the interaction of neutrophils, macrophages, and DCs with LECs, especially macrophages, is important for the progression of lymphedema. Below, we summarize the previously reported roles of different cell types in lymphedema, including several signaling pathways, therapeutic measures, and pathological processes (Figure 2).

### 4.1 Th1

Th1 cells play a critical role in the formation and progression of secondary lymphedema through their proinflammatory effects. S1PR1-deficient LECs promote the differentiation of CD4^+^ T cells into Th1 cells via direct contact (Kim et al., 2023). Similarly, in individuals with filarial lymphedema, BmA significantly increases the production of the Th1-type cytokines IFN-γ and TNF-α (Babu et al., 2009). Paradoxically, in a study of parasitic infection causing lymphedema, Babu S et al. reported a significant reduction in IFN-γ and TNF-α production by Th1 cells, with low expression of T-bet and attenuated Th1 responses (Babu et al., 2006). Loghry HJ et al. reported that Bma-LEC-2, which is secreted by parasites infecting patients with lymphatic filariasis, can selectively induce the apoptosis of Th1 cells and inhibit the host’s Th1 immune response, leading to persistent parasitic infection and the exacerbation of lymphedema disease (Loghry et al., 2022). Studies of Th1 cells in lymphatic filariasis-induced lymphedema still need further investigation. Similarly, Miyazaki T reported that abnormal activation of the calmodulin system in LECs inhibited the generation of TGF-β1, a process that affects the stability of Tregs and may indirectly affect the proinflammatory response of Th1 cells (Miyazaki et al., 2023). The ability of hyaluronidase and P-selectin to improve lymphedema differentiation by enhancing Th1 cells and reducing Th2 cell differentiation is promising (Kim et al., 2023; Cho et al., 2017). It can be speculated that increased Th1 cell differentiation exacerbates the development of lymphedema in the presence of blocked or dysfunctional lymphangiogenesis.

### 4.2 Th2

Th2 cells, a type of helper T cell, mainly secrete cytokines such as IL-4 and IL-13 during the progression of secondary lymphedema. Compared with Th1 cells, Th2 cells play an equally important role in the pathological process of lymphedema and in antifibrotic therapy. In pathogenesis, Savetsky IL et al. reported that IL-4 and IL-13 secreted by Th2 cells inhibit their proliferation, migration and tubular structure formation by promoting the apoptosis of LECs (Savetsky et al., 2015). In the case of lymphedema, impaired S1P signaling in LECs normally fails to regulate Th2 cell differentiation, further aggravating the symptoms of secondary lymphedema (Kim et al., 2023). Abnormal activation of the calmodulin (calpain) system of LECs leads to impaired stability of Th2 cell subsets, affecting the function of the lymphatic system (Miyazaki et al., 2023). A study by Avraham T et al. and Mehrara BJ et al. revealed that the inhibition of Th2 differentiation with IL-4- or IL-13-blocking agents, such as neutralizing antibodies, significantly relieved the symptoms of secondary lymphedema (Avraham et al., 2013; Mehrara et al., 2021).

### 4.3 Th17

Th17 cells in lymphedema and elephant cutaneous tumor symptoms due to secondary lymphatic filariasis are closely associated with Th17 cells (Fu and Liu, 2023). The immune response in lymphedema patients progresses in the directions of Th1, Th2, and Th17 (Pfarr et al., 2009). Among them, the levels of the inflammatory cytokines IL-17 and IFN-γ secreted by Th17 cells are significantly greater than those secreted by control cells (Anuradha et al., 2014). Th17 cells often aggravate secondary lymphedema by promoting inflammation and fibrosis progression. Although the relationship between LECs and Th17 cells has not been directly discussed in detail in lymphedema, Th17 cells are also a subset of CD4^+^ T cells, and it can be speculated that LECs may affect the differentiation or function of Th17 cells through a similar mechanism. In collagen-induced arthritis (CIA), the autophagy process of LECs modulates Th17 efflux from the lymph nodes (LNs), a process associated with SphK1 changes resulting in the S1P gradient (Harlé et al., 2021). Current studies on the interaction between Th17 cells and LECs involved in the pathogenesis of lymphedema are still scarce and warrant further study and discussion.

### 4.4 Th22

Few studies of Th22 cells in lymphedema are available, and the relationship between LECs and Th22 cells has not been discussed in detail. Only one study reported that Th22 cells from patients with filarial lymphedema coexpressed higher levels of IL-22 and IFN-γ than did those from controls (Anuradha et al., 2014). Th22 and Th17 cells are often involved in chronic inflammatory diseases. Therefore, it is important to explore the effects of Th22 cells on LECs in patients with lymphedema. Moreover, focusing on the role of Th17 and Th22 in patients with lymphedema is highly valuable for further understanding the pathogenesis of lymphedema.

### 4.5 Tregs

The interaction between Tregs and LECs plays an important role in the drainage of lymphatic fluid and the migration of immune cells. Treg numbers are significantly increased in secondary lymphedema tissues in both mice and humans (Gousopo et al., 2016a). Tregs promote the permeability of LECs and enhance the ability of other immune cells to span LECs through LTα1β2-LTβR signaling (Piao et al., 2020). In contrast, LECs are able to regulate PD-L1 migration and inhibit the inflammatory progression of lymphedema by binding to PD-L1 on Tregs (Piao et al., 2022). Importantly, Tregs are involved in the suppression of Th1/Th2 immune responses and the progression of tissue fibrosis to improve the function of lymphatic vessels (Gousopo et al., 2016b). Gkountidi AO et al. reported that IFN-γ upregulated the expression of MHC II in LECs and that MHC II presented antigens to Tregs to inhibit the activity of effector T cells and promote tumor growth (Gkountidi et al., 2021). Although the above reports were conducted in the context of tumor research, the immunomodulatory function of LECs and the interaction of Tregs may also have some reference value in the pathological process of lymphedema. In particular, the role of LECs in immune regulation may provide indirect implications for further understanding the immunological mechanisms of lymphedema.

### 4.6 Neutrophil

Secondary lymphedema promotes the infiltration of neutrophils and other inflammatory cells, exacerbating tissue fibrosis and chronic inflammatory responses (Zampell et al., 2012). Similarly, the degree of tissue inflammation in an obesity mouse model of lymphedema is strongly associated with increased infiltration of neutrophils (Savetsky et al., 2014). This process is associated with the possible secretion of chemokines (CXCL1/2/3/5) by LECs to attract neutrophils into lymphatic vessels and eventually into draining lymph nodes (dLNs). The synergistic effect of IL-17A and TNF-α promoted the secretion of chemokines by LECs (Ni et al., 2024). Moreover, the site of edema in patients with secondary lymphedema can be accompanied by neutrophilic skin lesions such as Sweet syndrome, which is also associated with the aggregation and infiltration of neutrophils (Guyot-Caquelin et al., 2010). Frueh FS et al. speculated that neutrophil infiltration in the lymph node area is associated with the pathogenesis of lymphedema after ischemia/reperfusion injury (Frueh et al., 2020). However, the interaction between LECs and neutrophils in lymphedema has not been explored in detail in the literature. Further studies on the interaction between LEC and neutrophils are highly valuable for studying the pathogenesis of lymphedema.

### 4.7 Macrophage

The interaction of LECs and macrophages is very important in the pathological process of secondary lymphedema (Lala et al., 2018; Ji, 2007). With chemokines secreted by LECs, M2 macrophages secrete VEGF-A, VEGF-C and VEGF-D and regulate CD4^+^ T-cell accumulation and Th2 differentiation to increase lymphangiogenesis and reduce fibrosis. Therefore, promoting macrophage-to-M2 macrophage polarization in tissues with secondary lymphedema may also be an effective therapeutic modality. (Lala et al., 2018). For example, Zhou Z et al. reported that substance P, a neurotransmitter, downregulates the NF-κB/NLRP3 pathway to polarize bone marrow-derived macrophages (BMDMs) into M2 macrophages, thereby alleviating secondary lymphedema (Zhou et al., 2023). Macrophages also play an important role in the pathogenesis of infectious lymphedema. Weinkopff T et al. reported that lymphedema triggered by filarial infection is associated with excretory secretions such as VEGF-A and IL-8, which stimulate macrophages to produce lymphangiogenic factors capable of causing lymphangiectasia to cause secondary lymphedema (Weinkopff et al., 2014). Research on macrophages in lymphedema has focused mainly on how macrophages act with LECs to participate in the generation of lymphatic vessels and the development of lymphedema. Related studies have focused on the roles of macrophages and LECs in signaling pathways, information molecules, and inflammatory mediators. However, the role of macrophages in the pathogenesis of lymphedema is still unclear and requires further study.

### 4.8 Dendritic cells

There are no reports on how dendritic cells (DCs) interact with LEC in lymphedema. Almost all studies have focused on how DCs influence LECs to participate in the regulation of lymphoid tissue inflammation, lymphatic vessel development, and lymphatic permeability. On the basis of the role of DCs in the pathological process of abnormal lymphatic vessels, it can be speculated that abnormal development and functional inactivation of the lymphatic system are closely associated with the occurrence of lymphedema. LECs are also able to capture and present exogenous antigens via MHC II to DCs, which are able to cross-present antigens to CD8^+^ T cells (Santambrogio et al., 2019; Kedl et al., 2017). In contrast, the secretion of factors such as TGF-β1 by LECs suppresses the maturation of DCs to reduce inflammation in lymphoid tissues (Christiansen et al., 2016). DCs play an important role in promoting interactions with LECs during the development of lymphoid tissues. Upon IFN-γ stimulation, DCs significantly increase the release of VEGF-C and act on LECs to promote the generation of lymphatic vessels (Gagliostro et al., 2016). Moreover, VEGF-C secreted by DCs enables LECs to secrete chemokines such as CCL21, and CCL21 binds to receptors on DCs such as CCR7 to guide DCs into lymphatic vessels (Permanyer et al., 2018). Furthermore, LECs support the migration of DCs from peripheral tissues to draining lymph nodes by expressing adhesion molecules such as CD112 (Haghayeg et al., 2024). In terms of regulating permeability, DCs regulate lymphatic vessel permeability through specific signaling pathways, such as the CCR7/IRF4 axis and LECs interaction (Permanyer et al., 2018). In this section, we focus on relevant studies and reports regarding the interaction between DCs and LECs, particularly within the inflammatory tissue of lymphatic vessels, with the goal of providing insights into the pathogenesis of lymphedema and its progression.

### 4.9 Fibroblasts

The fundamental pathology of secondary lymphedema, a significant complication of cancer treatment, is characterized by an imbalance between TGF-β1-mediated fibrosis and lymphangiogenesis (Baik et al., 2022). On the one hand, significant activation of the Smad2 signaling pathway in lymphedema-associated fibroblasts (LAFs) has been shown to induce epithelial‒mesenchymal transition (EMT) and promote the formation of α-smooth muscle actin-positive stress fibers and collagen deposition under specific conditions (Will et al., 2024a). A recent study revealed the critical role of an interactive network of fibroblasts and LECs in the tumor microenvironment in the progression of lymphedema. In patients with cholangiocarcinoma (CCA) who have lymph node metastasis (LNM), cancer-associated fibroblasts (CAFs) promote lymphangiogenesis by activating the ERK1/2-JNK pathway in LECs through the secretion of PDGF-BB (Yan et al., 2024). Furthermore, the recruitment and 3-D assembly of LECs are induced by fibroblasts stimulated with PDGF-D, resulting in increased LEC monolayer permeability (Cadamuro et al., 2019). Oral CAFs have been shown to promote LEC proliferation, migration, invasion, and lumen formation to a greater extent than normal fibroblasts (NFs) do (Chen et al., 2015). In summary, the interaction between fibroblasts, particularly CAFs, and LECs plays a significant role in the regulation of lymphangiogenesis. This cellular communication significantly influences remodeling of the tumor microenvironment and offers novel insights into the mechanisms underlying secondary lymphedema. While the majority of current studies have focused on CAF‒LEC interactions in the context of tumors, these findings highlight potential therapeutic targets for interventions aimed at regulating fibroblast function to either promote or inhibit lymphangiogenesis, thereby addressing the occurrence and development of lymphedema. Therefore, an in-depth study of the communication mechanisms between fibroblasts and LECs may lead to new strategies and direct therapeutic approaches.

### 4.10 Adipose-derived stem cells

The pathomechanism of lymphedema involves not only interstitial fluid accumulation, but also multidimensional changes such as chronic inflammation, fibrosis, and abnormal fat deposition (Burton et al., 2023). Notably, adipose tissue has a bidirectional relationship with the lymphatic system, in which obesity has been shown to be an independent risk factor. Recent studies have indicated that adipose-derived stem cells (ADSCs) may hold promise in the treatment of lymphedema, with their therapeutic effects potentially manifesting through the activation of lymphatic vessel neogenesis. *In vitro* investigations have demonstrated that ADSCs significantly promote the proliferation, migration, and tubular structure formation of human dermal LECs (Saijo et al., 2019). However, the direct effect of ADSCs on LECs remains to be elucidated. A study by Takeda K revealed that factors secreted by ADSCs were more effective than recombinant human vascular endothelial growth factor-C in inducing LECs proliferation, migration, and tube formation (Takeda et al., 2015). However, a significant gap remains in our understanding of the intricate cellular communication between adipocytes and LECs. To address this critical knowledge gap, it is imperative to undertake a systematic analysis of the underlying mechanisms governing the interaction between adipocytes and LECs. This analysis should be facilitated by the establishment of an *in vitro* model that enables coculture of adipocytes and LECs. This model not only provides novel insights into the complex interactions between these cell types but also serves as a valuable platform for the development of novel therapeutic strategies for lymphedema, with a specific focus on adipose metabolism.

## 5 Maintenance and dynamic regulation of the lymphatic system microenvironment

### 5.1 Cell junctions

#### 5.1.1 Connexins

Gap junctions (GJs), which are intercellular channels composed of connexin (Cx) subunits, enable cell-to-cell communication through the direct cytoplasmic exchange of ions and small molecules (Söhl and Willecke, 2004) (Table 3). Among these cell junctions, connexins (Cxs), GJ proteins, have been shown to play a key role in lymphoid development in vertebrates. Many studies suggest that Cx-associated lymphedema is caused by asynchronous contraction of the lymphatic vessels (Castorena-Gonzalez et al., 2018). The absence of different Cxs, including Cx 26, Cx 37, Cx 43, and Cx 47, has also been confirmed in many studies of lymphedema. For example, loss of expression of GJB2, encoding Cx26, suppresses peripheral lymphangiogenesis and causes severe lymphedema in mouse models (Dicke et al., 2011). In addition, Ostergaard P et al. and Ferrell RE et al. reported that mutations in the GJC2 gene encoding Cx47 were associated with hereditary primary lymphedema (Ferrell et al., 2010; Ostergaard et al., 2011). A study by Finegold DN revealed a positive association between the risk of secondary lymphedema after breast cancer treatment (Finegold et al., 2012). Brice G et al. confirmed through Sanger sequencing that a new mutation in the GJA1 gene encoding the Cx43 mutation an leads to oculodentodigital syndrome and primary lymphedema (Brice et al., 2013). Cx43 has a limited role in the lymphatic endothelium, and one study using a knockout mouse model revealed that although Cx43 is widespread in the endothelial layer of lymphatic vessels, its absence does not affect rhythmic contraction or calcium signaling synchronization in lymphatic vessels. It is Cx45, not Cx43, that truly regulates contraction rhythms (Castorena-Gonzalez et al., 2018). Davis MJ and colleagues first reported that the expression of CX45 in mouse lymph endothelial cells is essential for the normal function of the lymphatic system. Unfortunately, there are no reports of human lymphedema associated with Cx45 (GJC1) mutations. This may be because loss-of-function mutations in human Cx45 are potentially lethal and are thus not detected in primary lymphedema clinical screening through whole-exome sequencing (Davis et al., 2024). Kanady JD et al. reported that FOXC2 and Cx37 jointly control lymph valve development (Kanady et al., 2015). In addition, defects in Cx37 and Cx43 have been shown to cause primary lymphedema and chylothorax (Kanady et al., 2011). We currently have limited knowledge of the functional relationships and signaling pathways of Cxs with other proteins. At present, the study of the interaction between Cxs and other proteins can provide a deeper understanding of developmental disorders and the missing functions of LECs, establishing a basis for further exploration of the pathogenesis of lymphedema and therapeutic measures.

**TABLE 3:** Cell junctions in lymphedema.

Types of lymphedema	Category	Mechanisms of action	Data sources
Primary	Cx 26 (GJB2) (Dicke et al., 2011)	Related to lymphatic vessel formation	Mouse model
Primary and secondary	Cx 37 (GJA4) (Kanady et al., 2015; Kanady et al., 2011; Hadizadeh et al., 2018)	Related to lymphatic vessel growth and lymphatic valve development Mutations that are associated with secondary lymphedema in breast cancer patients after surgery	Mouse model Patient
Primary	Cx 43 (GJA1) (Castorena-Gonzalez et al., 2018; Brice et al., 2013)	Mutations that cause abnormal valve development and/or reduced valve density leading to lymphatic transport disorders	Mouse model Patient
Primary and secondary	Cx 47 (GJC2) (Ferrell et al., 2010; Ostergaard et al., 2011)	Mutations that cause abnormal valve development and/or reduced valve density leading to lymphatic transport disorders Mutations that are associated with secondary lymphedema following breast cancer treatment	Mouse model Patient
Nothing found	Cx45 (GJC1) (Davis et al., 2024)	Essential for lymphatic valve function Cx45 loss-of-function mutations may be lethal	Mouse model
secondary	ROCK2/JAM-A (Lee et al., 2023)	Specific knockdown of ROCK2 in the lymphatic system can reverse lymphedema	Mouse model LECs
Primary	Cadherin 4 (FAT4) (Betterman et al., 2020)	Mutations in calmodulin FAT4 cause Hennekam syndrome, one of the characteristics of which is lymphoedema	Patient
Primary	VE-cadherin (Yang et al., 2019; Cha et al., 2016)	VE-cadherin can bind to β-catenin, which is essential for the development of lymphatic valves	Mouse model
Nothing found	Claudin 5 (Frye et al., 2020; Mahamud et al., 2019)	The localization and stability of CLDN5 may affect lymphatic vascular permeability	Mouse model HLECs

#### 5.1.2 JAM-A

Junctional adhesion molecules (JAMs) are a family of adhesion molecule glycoproteins that are expressed on human LECs. There is a paucity of literature on the association between JAM and lymphedema (Ueki et al., 2008). Lee et al. successfully induced lymphedema by the cytokine-induced ROCK2/JAM-A complex in a 3D bionic model of lymphatic vessels. Lymphatic-specific knockdown of ROCK2 reverses lymphedema *in vivo* and could serve as a potential therapeutic target for lymphedema (Lee et al., 2023). The role of JAM in the pathogenesis and treatment of lymphedema deserves further investigation and discussion.

#### 5.1.3 Cadherin

VE-cadherin-mediated cellular connectivity and signaling regulatory networks have been demonstrated to play central roles in lymphedema development (Hägerling et al., 2018; Frye et al., 2015). VE-cadherin has been shown to bind to β-catenin, thereby maintaining Prox1, FOXC2, and GATA2 transcriptional regulation, which is required for lymphatic valve development (Yang et al., 2019; Cha et al., 2016). In addition, VE-cadherin was found to regulate valve formation through VEGFR2/3-AKT signaling and to synergistically maintain endothelial junction integrity with uPARAP (Coon et al., 2015; Gucciardo et al., 2025). GATA2, a key transcription factor, not only regulates VE-cadherin and claudin 5 (CLDN5) expression, but also its own expression is regulated by β-catenin signaling, suggesting that a bidirectional regulatory mechanism may be involved in this pathological process (Mahamud et al., 2019). Notably, mutations in the atypical calcineurin protein FAT4 have been identified in patients with Hennekam syndrome, which is characterized by lymphedema as one of its features. This finding underscores the critical role of FAT4 in the lymphovascular system (Betterman et al., 2020). Although the direct relationship between cadherins and lymphedema is not fully understood, recent studies have begun to reveal changes in cadherin localization and molecular expression that may act as potential triggers for lymphedema. For example, VE-cadherin-dependent signaling is critical for lymphatic valve formation and maintenance, and therapies that enhance downstream pathways have the potential to treat lymphedema. Therefore, future studies should investigate the specific role of cadherins in the pathogenesis of lymphedema to identify new intervention targets that could advance the early diagnosis and treatment of this disease.

#### 5.1.4 Claudin

Claudin 5 (CLDN5), a pivotal molecule in cell junctions, is regulated by both GATA2 and EphrinB2/EphB4 signaling, suggesting a potential role in edema formation through its impact on lymphatic endothelial barrier function. GATA2 mutations disrupt cellular junctions by downregulating CLDN5 and VE-cadherin. In contrast, the EphrinB2/EphB4-Rac1/Rho pathway has been demonstrated to dynamically regulate the localization and stability of CLDN5, suggesting that targeting this pathway may selectively regulate lymphatic vessel permeability (Frye et al., 2020; Mahamud et al., 2019). Although there is limited evidence for a direct association between CLDN5 and lymphedema, its aberrant expression has been recognized as a potential pathogenetic factor. In the future, the spatiotemporal regulatory network of CLDN5, as well as the mechanism of synergistic maintenance of the lymphatic barrier with VE-cadherin, must be analyzed, which will provide a basis for precise intervention.

### 5.2 Oxidative stress in lymphedema

Oxidative stress has been demonstrated to affect normal lymphatic function and exacerbate secondary lymphedema. Aging has been demonstrated to reduce the level of antioxidant enzymes (SODs) and increase superoxide anion levels in lymphatic vessels, thereby promoting chronic inflammation and weakening lymphatic vessel contractility (Pal et al., 2017). The level of oxidative stress in lymphedema is significantly elevated in pathological states. The accumulation of substantial quantities of lymphatic fluid within the affected tissues can result in local tissue hypoxia, which, in turn, can trigger excessive oxidative stress, thereby establishing a self-perpetuating cycle (Siems et al., 2002; Tabibiazar et al., 2006). The inhibition of cyclooxygenase (COX) has been demonstrated to significantly reduce edema and lower free radical levels in a mouse tail lymphedema model, suggesting the potential of antioxidant strategies in lymphedema treatment (Chang et al., 2013). In clinical trials, sodium selenite, an antioxidant, has been shown to reduce ROS production and mitigate symptoms of lymphedema. Furthermore, the observation of enhanced serum markers of oxidative stress in patients undergoing lymphatic venous anastomosis (LVA) and vascularized lymph node flap transfer supports the efficacy of alleviating oxidative stress in reducing lymphatic loading (Yang et al., 2021b). Consequently, interventions that target oxidative stress, such as the utilization of antioxidants and the enhancement of oxidative stress levels after surgical intervention, may offer novel approaches for the management of lymphedema. These interventions are also clinically significant, particularly in reducing the lymphatic load and enhancing lymphatic function.

## 6 Treatment

The management of lymphedema, a complex chronic disease, continues to face significant challenges, particularly in the context of primary lymphedema due to delayed diagnosis and heterogeneous progression patterns. The clinical management of secondary lymphedema mainly follows a framework based on core strategies, including complete decongestive therapy (CDT) and microsurgical techniques such as lymphovenous anastomosis (Table 4). However, the evidence supporting the long-term efficacy of these interventions remains to be thoroughly substantiated (Senger et al., 2023). In the context of secondary lymphedema, although recombinant VEGF-C and AAV gene therapies have been shown to significantly improve edema in animal models, their clinical application is limited by targeted delivery efficiency and high cost (Szuba et al., 2002; Tammela et al., 2007; Leppäpuska et al., 2022). In contrast, small-molecule drugs such as second-generation tetracyclines exhibit strong clinical translational potential through the dual mechanism of activating LECs proliferation and inducing M2 macrophage polarization (Furlong-Silva et al., 2021). Furthermore, the topical application of pirfenidone (PFD) has been demonstrated to inhibit TGF-β1 signaling, thereby reducing the degree of fibrosis and preventing the development of lymphedema (Baik et al., 2022). However, the utilization of these medications can result in a range of adverse effects, including gastrointestinal reactions and lipid metabolism disorders. Among natural products, Cynanchum atratum extract CAPW-1 promotes lymphangiogenesis through the TLR4/NF-κB pathway and has a synergistic effect with VEGF-C (Li et al., 2024). S R Narahari argued that the “integrative medicine” model promoted in India, which combines traditional and modern approaches and focuses on the quality of life of the patient, is an effective, low-cost way of managing lymphedema and could serve as a model for disability prevention of chronic diseases in developing countries (Narahari and Ryan, 2020). He also provided clear clinical evidence that an integrative medicine program integrating Ayurveda, yoga, anti-infective measures and compression therapy is effective in improving lymphedema, reducing infections, and improving quality of life in patients with lymphatic filariasis and that it is low cost and suitable for gRAS-root dissemination (Narahari et al., 2023). The combination of the ultramicrosurgical technique and ICG lymphangiography has yielded substantial limb volume reduction in postoperative patients. The underlying mechanism of this efficacy is closely associated with oxidative stress modulation (Will et al., 2024b). Future research endeavors must integrate single-cell genomics to elucidate the heterogeneity of LECs, develop engineered biomaterials, and construct AI predictive models to optimize individualized treatments. Ultimately, the aim of these efforts is to achieve a paradigm shift from symptom management to pathological reversal through multitarget regulatory networks.

**TABLE 4:** The related treatments for lymphedema.

Type of treatment	Specific interventions	Mechanism of action	Type of application	Dominance	Limitations
Traditional core therapy	Complete CDT (Senger et al., 2023)	Physical reduction (multilayer bandage/massage)	Primary secondary	International gold standard with clear short-term results	Insufficient evidence of long-term efficacy, lifelong maintenance therapy required
Microsurgical technique	Lymphatic vascular anastomosis (LVA) (Yang et al., 2021b)	Establishment of lymphatic bypass drainage	Secondary	Precision repair with small incisions	Dependent on operator experience; long-term results to be proven
Biological preparation	Recombinant VEGF-C/AAV gene therapy (Szuba et al., 2002; Tammela et al., 2007; Leppäpuska et al., 2022)	Promotes lymphatic neovascularization	Animal model	Significant improvement in edema	Inefficient targeted delivery; costly
Small-molecule drug	Second generation tetracyclines (Furlong-Silva et al., 2021)	Activates LEC proliferation and induces M2 macrophage polarization	Secondary	Dual mechanism with strong potential for clinical translation	Gastrointestinal reactions, lipid metabolism disorders and other side effects
	Topical application of PFD (Baik et al., 2022)	Inhibition of TGF-β1 signaling pathway, anti-fibrosis	Prophylactic	Stopping the progression of fibrosis	Skin irritation, photosensitivity
Natural product	CAPW-1 (Li et al., 2024)	TLR4/NF-κB pathway promotes lymphangiogenesis + synergizes with VEGF-C	Experimental research	Natural source, multitarget synergy	Lack of clinical data
Integrative medicine	Ayurvedic herbs/yoga/step pressure therapy (Narahari and Ryan, 2020; Narahari et al., 2023)	Integrated physical and phytotherapy	Resource-limited areas	Low cost, reduced infections, improved quality of life	Difficulty in standardization, wide individual variation in efficacy
Technology innovation portfolio	Ultra microscopy + ICG lymphography (Will et al., 2024b)	Precision targeting + oxidative stress regulation	Secondary	Significant limb volume reduction	High technical requirements, high equipment dependency

## 7 Conclusion and discussion

Lymphedema is a complex chronic disease, and its pathological mechanism involves genetic variations, signaling pathway abnormalities, dysregulated intercellular communication, immune microenvironmental disorders, and oxidative stress imbalance at multiple levels. This review methodically summarizes the molecular regulatory network of lymphedema-related signaling pathways and reveals the central roles of key pathways, such as the RAS/MAPK, PI3K/AKT, VEGF-C/VEGFR-3, and HGF/MET pathways, in the development, functional maintenance, and pathological damage of lymphatic vessels. For example, aberrant activation of the RAS/MAPK pathway has been closely associated with hereditary lymphedema, such as NS. Conversely, dysregulation of the VEGF-C/VEGFR-3 axis has been identified as a significant driver of primary lymphedema, including Milroy disease. Furthermore, TGF-β1 signaling has been shown to exacerbate the progression of secondary lymphedema by promoting fibrosis and inflammatory responses. In addition, the vicious cycle of oxidative stress and chronic inflammation has been demonstrated to further deteriorate lymphatic function. Notably, mutations in GJ proteins, such as Cx37 and Cx47, have been shown to result in impaired lymphatic return by interfering with the synchronization of lymphatic vasoconstriction. This provides a novel perspective for understanding abnormal lymphatic vessel dynamics.

At the level of cellular interactions, the dynamic interactions of LECs with immune cells, such as Th1, Th2, Treg, and macrophages, constitute a central feature of the lymphedema microenvironment. For example, IL-4 and IL-13, which are secreted by Th2 cells, have been shown to inhibit lymphangiogenesis by inducing LEC apoptosis. In contrast, M2 macrophage polarization has been shown to promote compensatory lymphangiogenesis through the secretion of VEGF-C. These findings contribute to a more comprehensive understanding of the immunopathological mechanisms underlying lymphedema and establish a theoretical foundation for targeted immunomodulation as a therapeutic approach for this condition.

The existing therapeutic interventions for lymphedema and the current state of research in this domain are inherently limited and encumbered by significant challenges. Clinical interventions, including compression therapy and surgical procedures, are effective in mitigating symptoms. However, they do not address the underlying pathophysiology. Furthermore, there is a paucity of precise treatment regimens that are tailored to specific molecular typing profiles, which is crucial for the management of this condition. In the context of individual signaling pathways, single-target interventions may be inadequate for reversing the pathological process. Consequently, there is an imperative need to explore synergistic regulatory strategies that involve multiple pathways. However, notably, the majority of signaling pathways exhibit cross-talk, a phenomenon exemplified by PI3K/AKT and RAS/MAPK. This observation underscores the existence of substantial research potential and a notable gap in the existing knowledge. Moreover, the majority of extant studies are predicated on animal models, and the heterogeneity of primary versus secondary lymphedema may limit the generalizability of therapeutic strategies. By simulating lymphatic vessel–immune cell interactions, targeting antioxidant–promoting lymphatic regeneration, and remodeling the intralymphatic immune–metabolic microenvironment, further research into the mechanism of lymphedema and exploring therapeutic strategies are highly important. It is anticipated that this study will generate novel research concepts and insights relevant to targeted therapy for lymphedema.

In conclusion, it is imperative that research on lymphedema integrate the exploration of molecular mechanisms with the innovation of therapeutic modalities. In the future, the therapeutic bottlenecks currently experienced by lymphedema patients may be overcome through targeted regulation of oxidative stress, multipathway interventions, and precision medicine strategies. Such strategies may improve lymphatic function and reverse the pathological process, thereby improving the quality of life for these patients.
